# Tumor Necrosis Factor-Alpha (TNF-α) and Interleukin-6 (IL-6) Cytokines in the Injured Meniscus of Patients With Knee Subchondral Insufficiency Fractures: A Potential Association With Preoperative Pain

**DOI:** 10.7759/cureus.77734

**Published:** 2025-01-20

**Authors:** Hideaki Fukuda, Yoshihiro Sakuma, Kazuhide Inage, Kenji Takahashi, Ichiro Yamaura, Hideaki Shiratsuchi, Seiji Ohtori

**Affiliations:** 1 Sports Medicine and Joint Center, Inanami Spine and Joint Hospital, Tokyo, JPN; 2 Sports Medicine and Joint Center, Funabashi Orthopedic Hospital, Funabashi, JPN; 3 Department of Orthopedic Surgery, Graduate School of Medicine, Chiba University, Chiba, JPN; 4 Department of Orthopedic Surgery, Funabashi Orthopedic Hospital, Funabashi, JPN; 5 Department of Orthopedics, Chiba University Hospital, Chiba, JPN

**Keywords:** early-stage knee osteoarthritis, medial meniscal posterior root tear, pre-operative pain, proinflammatory cytokines, subchondral insufficiency fracture of the knee

## Abstract

Background

The development of subchondral insufficiency fracture (SIF) of the knee following a medial meniscal posterior root tear (MMPRT) results in pain and limitations in daily activities. Pain in patients with SIF is associated with local chronic inflammation in the knee joint, involving the production of inflammatory cytokines such as tumor necrosis factor-alpha (TNF-α), interleukin-6 (IL-6), and nerve growth factor (NGF). This study aimed to quantify the levels of proinflammatory cytokines (TNF-α, IL-6, NGF) in the injured medial meniscus (IMM) tissue of patients with knee SIF and examine their potential association with preoperative pain and functional scores.

Methods

Meniscus samples were collected from 17 patients with knee SIF (mean age: 62.8 ± 2.6 years) who underwent total knee arthroplasty. The tissue samples were categorized into two groups based on the degree of injury: the IMM and the non-injured healthy lateral meniscus (HLM) as the control group. The levels of TNF-α, IL-6, and NGF in both groups were measured using an enzyme-linked immunosorbent assay (ELISA), and differences were evaluated with the Wilcoxon signed-rank test. The association between preoperative functional and pain scores and the levels of each inflammatory mediator was analyzed using Spearman’s correlation.

Results

TNF-α and IL-6 were detectable in the meniscus tissues, while NGF levels were negligible. The levels of TNF-α and IL-6 were significantly higher in the IMM group compared to the HLM group (p<0.05). Furthermore, TNF-α and IL-6 levels in the IMM group were correlated with the pain subscale of the Western Ontario and McMaster Universities Osteoarthritis Index (WOMAC).

Conclusions

This study demonstrated a significant increase in TNF-α and IL-6 cytokine levels in the IMM compared to the non-injured healthy meniscus. These findings suggest that inflammatory mediators within the IMM may be associated with preoperative pain in patients with SIF in the medial femoral condyle resulting from MMPRT.

## Introduction

The meniscus is a semilunar fibrocartilaginous tissue that plays a critical role in knee stability, joint congruency, shock absorption, lubrication, proprioception, and nutrient distribution to the articular cartilage [1,2]. It plays an indispensable role in cartilage protection and the prevention of osteoarthritis (OA) in the knee joint [3]. Loss of the mechanical function of the meniscus increases the risk of OA [4-8], and subchondral insufficiency fracture (SIF) of the knee [9].

SIF, even in early-stage knee OA is characterized by a sudden surge of inflammation in the knee joint that often causes severe sharp pain. Several inflammatory mediators in the knee disease process have been reported, including tumor necrosis factor-alpha (TNF-α), interleukin-6 (IL-6), and nerve growth factor (NGF). However, few studies have evaluated proinflammatory cytokines in the injured meniscus and correlated proinflammatory cytokine levels with functional and pain scores among patients with knee pain [10,11].

An earlier study by the present authors reported level III evidence for the crucial role of local inflammatory mediators in the disease process of meniscal lesions, which might contribute to the development of cartilage damage [12]. Despite the significant effect of these mediators on knee OA or SIF, the specific mechanism through which inflammatory mediators are associated with meniscal tissue injury remains unclear. We hypothesized that high levels of specific inflammatory mediators, TNF-α, IL-6, and NGF, would be detected in the injured medial meniscus (IMM) of the knee joint and that these inflammatory mediators might be associated with the pre-operative Western Ontario and McMaster Universities Osteoarthritis Index (WOMAC) score.

## Materials and methods

This was a cross-sectional experimental study evaluating the association between inflammatory cytokines and preoperative pain. The data used were collected from surgeries performed between 2013 and 2016. All surgical procedures were performed at Funabashi Orthopedic Hospital, and all subsequent research was conducted at the Department of Orthopedic Surgery, Chiba University School of Medicine. The study was approved by the Funabashi Orthopaedic Hospital Institutional Review Board (approval number: 2022041).

Selection of participants

Patients evaluated at our hospital for knee pain, particularly limp-walking and nocturnal pain, between 2012 and 2016, who were diagnosed with subchondral insufficiency fracture in the medial femoral condyle (MFC) of the knee joint caused by a medial meniscus posterior root tear (MMPRT) based on physical examination, radiographs, and magnetic resonance imaging (MRI) were looked into as prospective participants. All patients showed early-stage medial knee OA (Kellgren-Lawrence grade 2) on X-ray, medial meniscal tear and extrusion, subchondral insufficiency fracture, and bone marrow edema in the MFC on MRI.

Patients who failed conservative treatment (protected weight bearing, non-steroidal anti-inflammatory drugs (NSAIDs), hyaluronic acid injections, and physical therapy) for three to six months on an outpatient basis were included in the study. Informed consent was obtained from all participants for the proposed surgery after explaining high tibial osteotomy (HTO), unicompartmental knee arthroplasty (UKA), or total knee arthroplasty (TKA). Patients were informed that if lesions, including bone marrow edema, involved more than 50% of the condylar surface, it would lead to condyle collapse, and therefore, arthroplasty would be required [9,13]. In addition, it was explained that the choice between TKA and UKA for SIF of the knee largely depended on factors such as the extent of knee damage and patient characteristics. TKA was generally recommended for SIF patients with persistent severe knee pain and widespread MFC damage, as it provided a comprehensive approach to knee joint replacement [14]. In contrast, UKA was considered for patients with isolated compartmental damage and less extensive OA. Patients with a history of previous surgery, concomitant ligament deficiency, lateral knee joint OA, lateral meniscus injury and degenerative changes, articular cartilage injury of the lateral compartment, or other pathologies (tumors, infections, coagulation disorders, inflammatory, rheumatic, and metabolic diseases) were excluded.

A total of 31 patients with SIF in the MFC of the knee joint after MMPRT underwent surgery. Regarding the surgical procedures, one case was HTO, eight cases were UKA, and 22 cases were TKA. All surgeries were performed by one experienced surgeon (HS). Of the 22 TKA cases, five patients were excluded for the following reasons: lateral meniscus injury or degenerative changes of the lateral meniscal tissue. A total of 17 patients (six male and 11 female patients; mean age 63.2 ± 3.0 years, range 58-68 years) who underwent TKA surgery were included in this study.

Surgical procedure and measurement

The injured medial meniscal tissue and healthy lateral meniscus (HLM) were resected according to routine procedures for arthroplasty. Each sample included at least three to four pieces of tissue obtained using an oval punch after resection. The most damaged lesions in MMPRT were harvested. Lateral meniscal tissues were harvested as the control group. For analysis, tissue samples were categorized into the following groups: IMM and non-injured HLM.

Meniscal tissue samples were immediately stored at −80°C. The samples were thawed, subsequently diced, and lysed with 500 μL of CelLytic™ M Cell Lysis Reagent (Merck Group, St. Louis, Missouri, United States). Inflammatory mediator quantification was performed using double-antibody sandwich enzyme-linked immunosorbent assays (ELISA) for TNF-α, IL-6 (R&D Systems, Inc., Minneapolis, Minnesota, United States), and NGF (Boster Bio, Pleasanton, California, United States), following the manufacturers' protocols.

The functional status and pain level of each patient were evaluated using the WOMAC score. The index consists of three subscales: pain, stiffness, and physical function. A higher score on the WOMAC scale represents poorer knee function or greater pain.

Statistical analysis

Differences in the concentration of inflammatory mediators (TNF-α, IL-6, and NGF) in tissue samples were evaluated using the Wilcoxon signed-rank test. Statistical differences between the two groups (IMM and HLM) were determined using the Mann-Whitney U test followed by Bonferroni's correction for multiple testing, and the statistical significance among the groups was determined using the Kruskal-Wallis test. The significance of correlations was determined by Spearman's rank correlation test. The association between pre-operative pain score and the level of each inflammatory mediator was evaluated using Spearman's correlation. The WOMAC was used to assess self-reported physical function, pain, and stiffness. A p-value < 0.05 was considered significant. All statistical analyses were performed with Stata Statistical Software: Release 13 (2013; StataCorp LLC, College Station, Texas, United States).

## Results

The study group consisted of six men and 11 women, with a mean age of 62.8 ± 2.6 years (range 58-68 years) with a mean BMI of 28.6 ± 3.3 (range 25.2-37.8) (Table 1). Regarding BMI, nine out of the 17 patients had a BMI exceeding 28, and three had a BMI over 30, indicating a high prevalence of patients with elevated BMI. Additionally, the International Cartilage Repair Society (ICRS) cartilage injury grade for the MFC showed that all cases were classified as grade 3 or higher, with the most severe grade 4 observed in seven patients. The WOMAC score, which comprises three subscales, pain, stiffness, and physical function, was assessed and recorded on the day prior to surgery (Table 2).

**Table 1:** The mean, standard deviation, and range of age, BMI, and WOMAC scores of the included patients (N=17) WOMAC: Western Ontario and McMaster Universities Osteoarthritis Index

Parameter	Mean ± SD	Range
Age (years)	62.1 ± 3.1	58 - 67
BMI (kg/m²)	28.6 ± 3.0	24.4 - 37.8
WOMAC Total Score	64.6 ± 8.6	51 - 77
Pain	15.9 ± 2.4	12 - 20
Stiffness	6.0 ± 1.4	3 - 8
Function	41.8 ± 9.4	27 - 52

**Table 2:** Demographic data of the participants (N=17) MFC: medial femoral condyle; WOMAC: Western Ontario McMaster University Osteoarthritis Index

Case	Age (years)	Sex	BMI (kg/m^2^)	Affected side	ICRS grade (MFC)	WOMAC total score	WOMAC Pain subscale	WOMAC Stiffness subscale	WOMAC Function subscale
1	61	M	24.4	Right	3A	53	14	6	33
2	67	F	33.2	Left	4	72	14	7	51
3	65	M	27.3	Left	4	77	20	6	51
4	61	M	27.8	Left	4	63	16	3	44
5	58	M	29.1	Left	4	58	16	8	34
6	63	F	34.6	Right	3B	74	16	8	50
7	64	M	37.8	Left	4	65	16	8	41
8	67	F	28.1	Left	4	70	14	6	48
9	59	F	27.5	Left	4	70	14	6	50
10	60	M	25.2	Right	3B	51	20	4	27
11	62	M	26.2	Right	3B	56	20	6	30
12	61	F	28.1	Right	4	52	14	8	30
13	63	F	28.1	Right	3A	72	14	7	51
14	62	F	26.6	Left	3B	58	20	4	34
15	68	F	27.1	Right	3A	72	15	6	51
16	67	F	28.1	Left	3B	70	14	6	52
17	67	F	28.1	Left	4	70	12	6	52

Concentrations of inflammatory mediators in the meniscus

**Figure 1:**
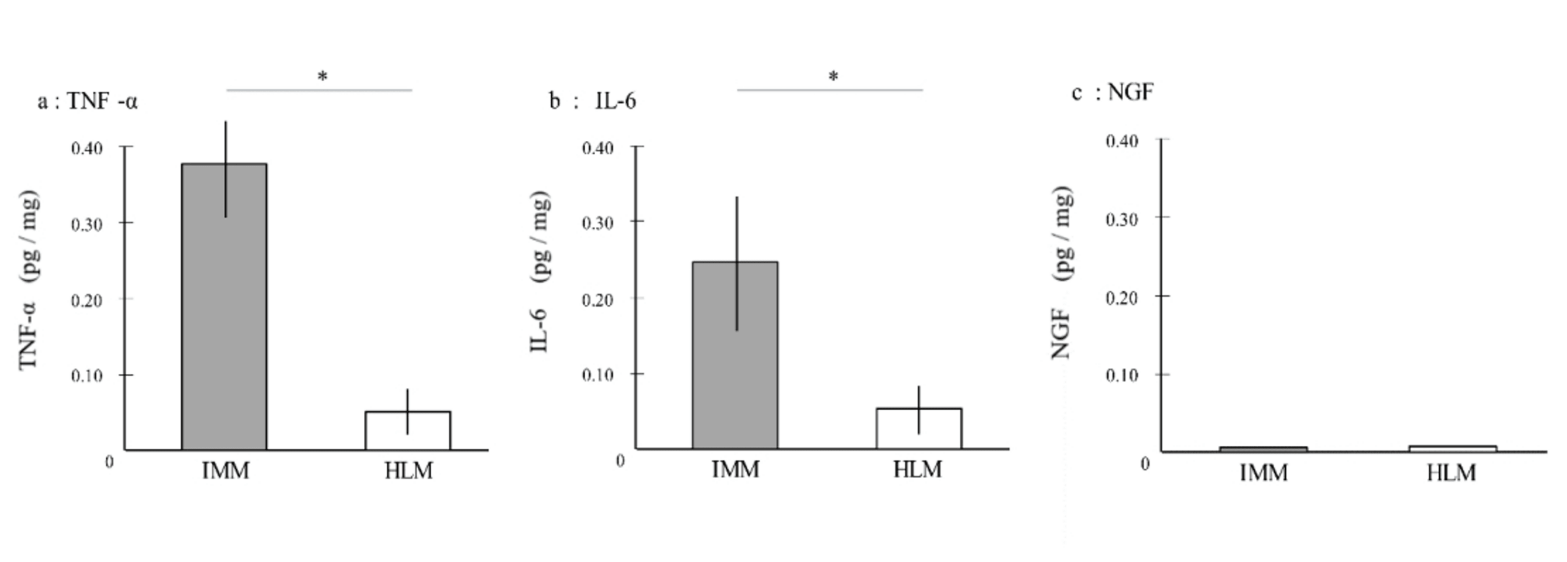
Levels of inflammatory mediators (TNF-α, IL-6 and NGF) in injured medial meniscus and non-injured healthy lateral meniscus tissues. Values are reported as a mean±SD * p < 0.05 (a) TNF-α levels (pg/mg) in IMM and HLM. TNF-α levels were elevated in the IMM tissue (IMM: 0.37 ± 0.11 pg/mg; HLM: 0.05 ± 0.03 pg/mg; p = 0.011); (b) IL-6 levels (pg/mg) in IMM and HLM. IL-6 levels were also elevated in the IMM tissue compared to the HLM tissue (IMM: 0.24 ± 0.11 pg/mg; HLM: 0.05 ± 0.02 pg/mg; p = 0.028); (c) NGF levels (pg/mg) in IMM and HLM. Levels of NGF were not sufficiently detectable in either group. Differences in the concentration of inflammatory mediators in tissue samples were evaluated using the Wilcoxon signed-rank test. Statistical differences between the two groups (IMM and HLM) were determined using the Mann-Whitney U test followed by Bonferroni's correction for multiple testing, and the statistical significance among the groups was determined using the Kruskal-Wallis test. TNF-α: tumor necrosis factor-alpha; IL-6: interleukin-6; NGF: nerve growth factor; IMM: injured medial meniscus; HLM: healthy lateral meniscus

Compared to the HLM tissue, TNF-α levels were significantly elevated in the IMM tissue (IMM: 0.37 ± 0.11 pg/mg; HLM: 0.05 ± 0.03 pg/mg) (p = 0.011). Similarly, IL-6 levels were higher in the IMM tissue compared to the HLM tissue (IMM: 0.24 ± 0.11 pg/mg; HLM: 0.05 ± 0.02 pg/mg) (p = 0.028). NGF levels were not sufficiently detectable in either group (Figure 1).

Correlation between WOMAC score and cytokine concentration

**Figure 2:**
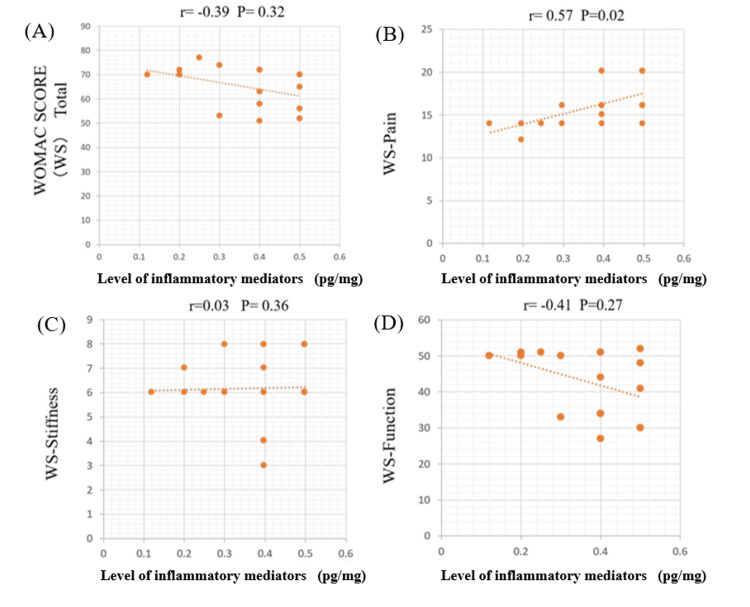
Correlations between TNF-α and the WOMAC score in the medial meniscus (MM) Each number corresponds to a specific subscale (total WOMAC score, pain, stiffness, and physical function). TNF-α demonstrated a significant positive correlation with the pain score (p < 0.05), while no correlation was observed with the total WOMAC score or the other subscales. TNF-α: tumor necrosis factor-alpha; WOMAC: Western Ontario and McMaster Universities Osteoarthritis Index

**Figure 3:**
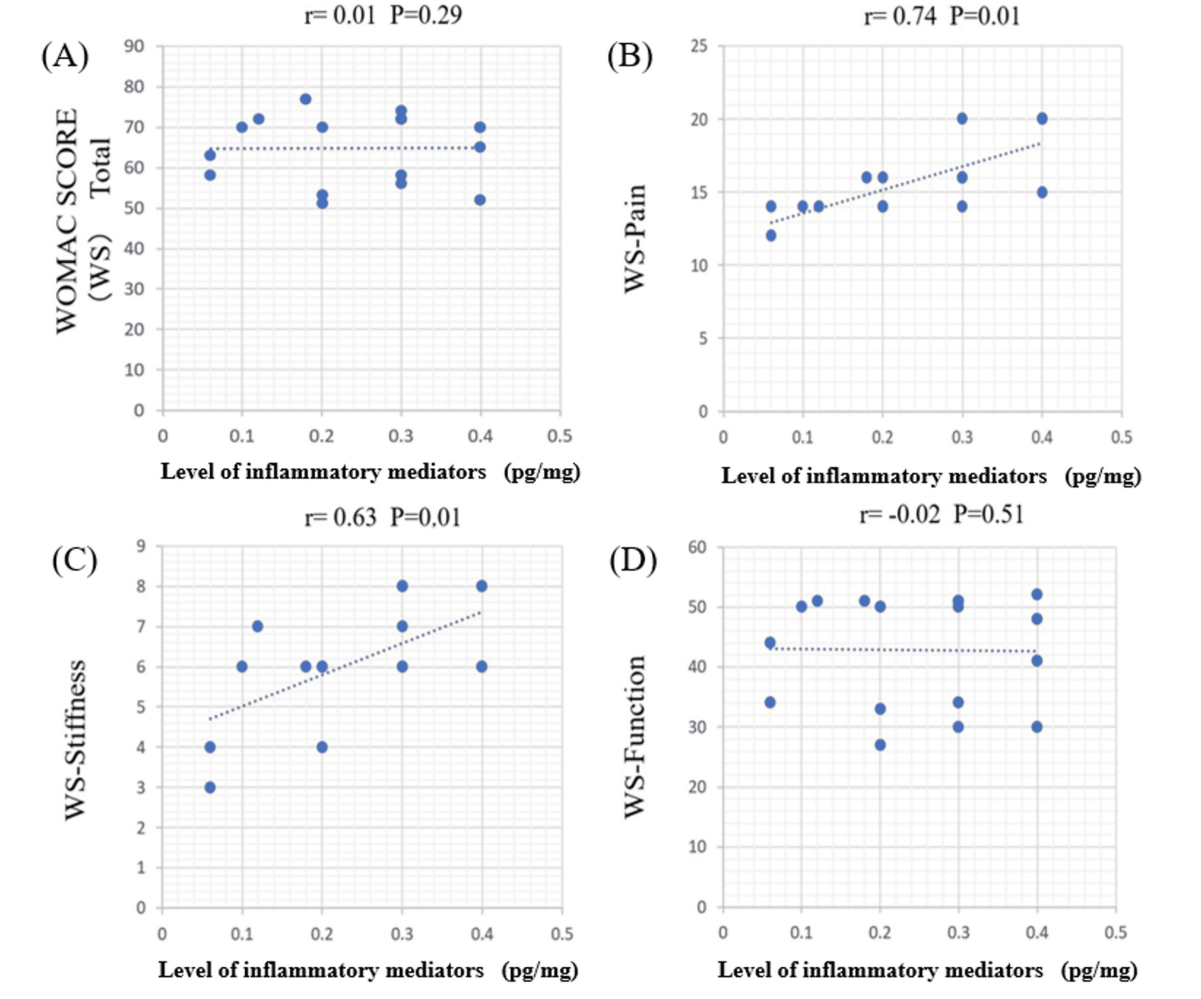
Correlations between IL-6 and the WOMAC score in the medial meniscus (MM) IL-6 showed a significant positive correlation with both the stiffness score (p < 0.05) and the pain score (p < 0.05). However, no correlation was observed with the total WOMAC score or the other subscales. IL-6: interleukin-6; WOMAC: Western Ontario and McMaster Universities Osteoarthritis Index

**Figure 4:**
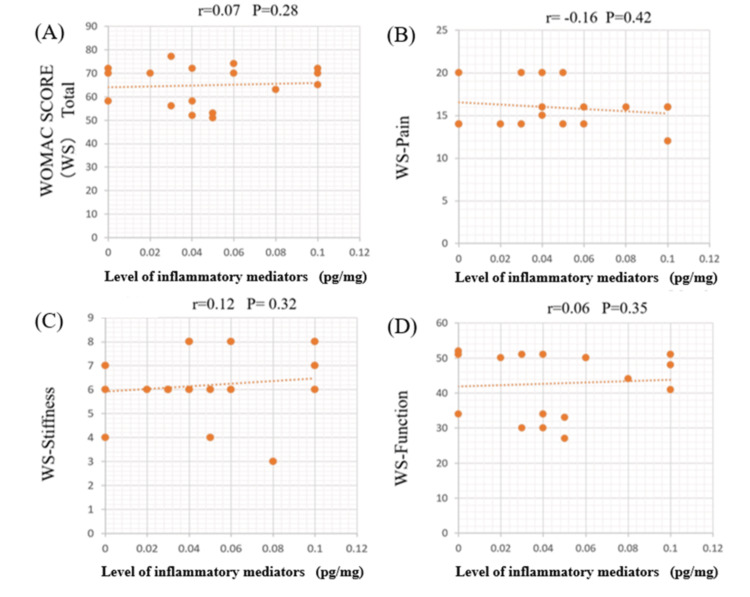
Correlations between TNF-α and the WOMAC score in the lateral meniscus (LM) TNF-α in the LM showed no correlation with the total WOMAC score or any of the subscales. TNF-α: tumor necrosis factor-alpha; WOMAC: Western Ontario and McMaster Universities Osteoarthritis Index

**Figure 5:**
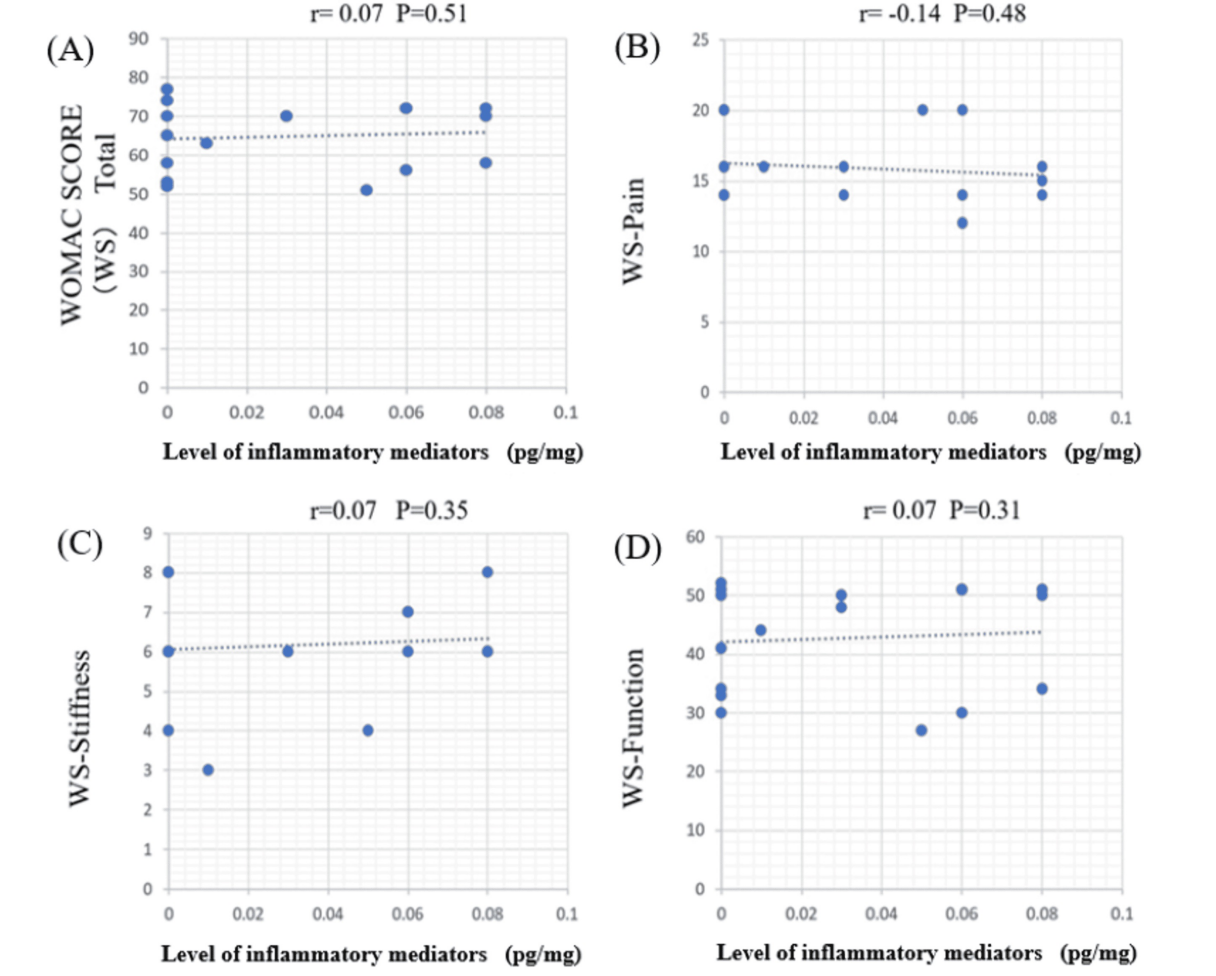
Correlations between the IL-6 and the WOMAC score in lateral meniscus (LM) IL-6 exhibited no correlation with the total WOMAC score and the other subscales. IL-6: interleukin-6; WOMAC: Western Ontario and McMaster Universities Osteoarthritis Index

Figures 2-5 illustrate the correlations between detectable cytokines and the WOMAC score. Group A represents TNF-α, and Group B represents IL-6.

## Discussion

The major finding of this study was the increased local inflammatory mediator levels in the IMM, which could be a key factor in pain and degeneration in comparison to the non-injured lateral meniscus. Additionally, it was suggested that there may be a potential association between the levels of inflammatory mediators and preoperative pain scores. Knee pain is commonly related to local chronic inflammation of the knee joints, which involves the production of inflammatory cytokines such as TNF-α, IL-6, and NGF. These inflammatory cytokines are believed to promote pathological OA and are present in synovial fluids and membranes. These proinflammatory cytokine mediators in the knee joint are known to contribute to knee OA by promoting cartilage degradation [10,11,15].

Although several mechanisms have been implicated in joint inflammation and pain, the contribution of injured menisci to these processes remains unclear. When the meniscus is damaged, it can lead to an inflammatory response in the knee joint. This inflammation results in the release of various cytokines, such as IL-1, TNF-α, and IL-6, into the joint's synovial fluid and synovial tissues. These inflammatory cytokines can exacerbate pain by sensitizing nerve endings in the joint, leading to heightened pain perception. Additionally, elevated levels of cytokines can contribute to the progression of joint degeneration, potentially leading to conditions like OA [12,16,17].

The relationship between meniscal pain and cytokines highlights the importance of managing inflammation to alleviate symptoms and slow down joint damage progression. To better understand this connection, measuring inflammatory cytokine levels in synovial fluid or synovial membrane tissue during surgery can help assess their contribution as confounding factors in knee pain and inflammation-related outcomes. Future studies should evaluate the correlation between the results of the present study and the results obtained from synovial fluids and synovial membranes.

There are a few reports that confirm the presence of these cytokines in the injured meniscus, and other studies have found increased IL-6 and IL-8 mRNA expression in the injured meniscus [18,19]. Furthermore, an earlier study by the current authors demonstrated that proinflammatory mediators produced by the injured meniscal tissues were contributing factors to a general inflammatory response in the knee joint [12]. The motivation for the present study stems from this previous study, in which three cytokines-TNF-α, IL-6, and NGF-in the synovial tissue of patients with meniscal injuries were targeted. As a continuation of that study [12], we are quantifying the same three cytokines in the IMM. However, focusing only on these three cytokines limits the scope of the study, which is one of its limitations.

Correlation between cytokine concentrations and the WOMAC score

The present study demonstrated that TNF-α derived from the IMM was significantly correlated with pain, while IL-6 showed a positive correlation with both pain and stiffness, which indicates that IL-6 primarily affects the progress of the degeneration of fibrocartilage in the meniscus that leads to joint stiffness. In contrast, cytokines derived from the non-injured HLM showed no correlation with WOMAC scores, pain, stiffness, or any other subscales. TNF-α initiates a cascade of inflammatory reactions through the production of ILs and directly activates sensory neurons via its receptors [20]. IL-6 has been reported to play a crucial role in OA pathogenesis by initiating inflammatory responses [21,22]. NGF, an inflammatory cytokine that specifically stimulates sensory nerve growth, is reportedly upregulated in human osteoarthritic chondrocytes and synovial fibroblasts, suggesting its vital role in OA pathophysiology [23-26]. The correlation between meniscal injury and OA has been established by many other studies, reporting meniscal injury as a risk factor for the development of OA. However, the biological effects of inflammation in the injured meniscus remain unclear. While there are some reports about IL-6 in the injured meniscus [18,25], little has been reported about TNF-α. No reports have been found that examine the relationship between the inflammatory cytokine TNF-α and preoperative pain scores.

Both the mechanical and biological pathways associated with meniscal injury must be understood to establish optimal therapy. In cases of SIF in the knee, meniscectomy alone is considered insufficient to relieve pain; therefore, osteotomy and arthroplasty have been reported as appropriate treatment options [27]. However, in cases of only painful meniscus injuries, meniscectomy might still be an option if the future progression to OA is understood, or injection therapy such as PRP to control pain cytokines may be an option.

The role of several inflammatory mediators in the progression of knee OA or SIF has been studied, including TNF-α, IL-6, and NGF. In addition, high concentrations of TNF-α and IL-6 in the joint fluid are associated with OA [28]. Despite the important contribution of these mediators to OA, the specific mechanism by which inflammatory mediators are associated with meniscal tissue injury is not determined. Some studies have reported elevations of TNF-α and IL-6 in joint synovial fluid after meniscus injury [3,16,17,28].

Although inflammatory responses in meniscal tissue alone were evaluated in this study, findings from previous studies are consistent with ours. Proinflammatory cytokines produced by injured meniscal tissue could be one of the origins contributing to a general inflammatory response of the knee joint after meniscal injuries. Turati et al. concluded that TNF-α levels were significantly (p < 0.05) greater in the bucket-handle tear group (BH) compared with the posterior horn tear group (PH), whereas IL-1β levels were significantly greater (p < 0.05) in the PH group compared with the BH group [29]. Both BH and PH groups were consistent in presenting a positive correlation between concentrations of IL-6 and IL-1β. A fundamental difference in IL-10 responsiveness between the two groups was noted; specifically, levels of IL-10 were positively correlated with IL-6 in the BH group, whereas in the PH group, levels of IL-10 were positively correlated with IL-1β. Their results suggested a possible influence of the meniscal tear pattern on the articular cytokine responsiveness. 

Orita et al. indicated that the concentrations of proinflammatory cytokines in the synovial fluid can be correlated with the WOMAC scores of patients with knee OA. The correlation between TNF-α and radiographic OA grading was not significant, whereas it had a significant correlation with WOMAC scoring. IL-6 levels had a significant negative correlation with KL grading and a significant correlation with the stiffness subscale. It concluded that these two cytokines were moderately correlated, and the results suggested that these cytokines play different roles in the pathogenesis of synovitis in the knee joint [15].

The present study, which sampled the meniscus instead of synovial fluid, also concluded that TNF-α is associated with preoperative pain. This research indicated that IL-6 in the IMM group exhibited a significant positive correlation with the stiffness subscale. The findings suggest that IL-6 within the damaged meniscus in the IMM group is associated with postoperative pain and joint dysfunction.

The current study confirmed the production of inflammatory mediators, especially TNF-α and IL-6, due to meniscal tissue injury, which could trigger inflammatory pain signals in patients with subchondral insufficiency fracture in the MFC of the knee joint after MMPRT. In addition, there was a significant correlation between preoperative WOMAC pain scores and the levels of TNF-α and IL-6. Specifically, when the meniscus is subjected to physical injury or wear, the resident cells, particularly fibrochondrocytes, become activated. As a result, inflammatory substances called cytokines are produced. IL-6 and TNF-α are representative pro-inflammatory cytokines that play a role in promoting immune system responses [20-22]. This process is thought to proceed similarly to that in articular cartilage, as follows: (i) Meniscus injury or stress: When the meniscus is damaged, mechanical stress is applied, resulting in cellular damage. Injury can also occur due to wear or trauma; (ii) Production of pro-inflammatory cytokines: Cells at the injury site trigger an inflammatory response, releasing cytokines such as IL-6 and TNF-α. These substances promote inflammation and recruit other immune cells, thereby supporting repair and defensive responses at the site of injury.

In contrast, NGF in the injured meniscal tissue was not detected in this study. The absence of an influence of meniscal injury on NGF could explain the absence of sensory nerve growth in the injured lesions. Cytokines act as nociceptive triggers and also induce the expression of other potentially nociceptive molecules and direct pain mediators, such as nitric oxide and prostaglandins. Other researchers demonstrated that inflammatory and pain mediators were upregulated in canine joints with spontaneous meniscal tears, and inflamed canine meniscus cells could contribute to joint inflammation and pain. In addition, the elevated expression of NGF detected in torn canine menisci is likely related to ongoing chronic inflammation in meniscal tears [30]. However, it is unclear whether the damaged meniscus plays an active role in NGF release. Further studies on the association between meniscal injury, inflammatory response, and knee pain are warranted.

Therapeutic implications of increased TNF-α and IL-6 levels

The findings of this study indicate that elevated levels of TNF-α and IL-6 in the IMM are significantly correlated with preoperative pain and stiffness, suggesting their potential as therapeutic targets for alleviating symptoms and preventing disease progression. This insight opens up several avenues for therapeutic interventions.

Anti-Cytokine Therapies

TNF-α inhibition: The strong correlation between TNF-α and pain highlights its role as a potential therapeutic target. Biologic agents like infliximab or adalimumab, which are currently used in inflammatory conditions such as rheumatoid arthritis [31,32], could be investigated for their efficacy in meniscus-related knee pain. These agents could help reduce pain by interrupting the inflammatory cascade initiated by TNF-α.

IL-6 blockade: The role of IL-6 in promoting stiffness and degeneration of the fibrocartilage suggests that IL-6 inhibitors like tocilizumab may have the potential to reduce stiffness and prevent further joint damage.

Intra-Articular Injection Therapies

Direct delivery of anti-inflammatory agents to the joint through intra-articular injections could provide localized symptom relief with minimal systemic side effects. For example: (i) Platelet-rich plasma (PRP): PRP contains anti-inflammatory components that may regulate the levels of cytokines like TNF-α and IL-6, potentially reducing pain and inflammation [33]; (ii) Hyaluronic acid with anti-cytokine additives: Enhanced formulations of hyaluronic acid that include anti-inflammatory agents could be developed to provide both mechanical lubrication and cytokine modulation.

Combination Therapies

Given the multifaceted roles of TNF-α and IL-6, combination therapies targeting multiple inflammatory pathways may prove to be more effective. For example, a combination of anti-TNF-α therapy with hyaluronic acid injections could address both inflammation and joint lubrication. Integrating physical therapy with biologic therapies may further reduce stiffness and enhance functional outcomes.

Personalized Medicine

The correlation between cytokine levels and WOMAC scores suggests that cytokines such as TNF-α and IL-6 could be used as biomarkers for personalized treatment plans. For example, patients with high levels of TNF-α might benefit more from anti-TNF-α therapy while patients with elevated IL-6 could be candidates for IL-6 blockade or stiffness-focused rehabilitation strategies.

Future research directions

To optimize these therapeutic strategies, future studies should investigate the efficacy of biologic agents targeting TNF-α and IL-6 in patients with meniscal injuries, evaluate the long-term outcomes of combining cytokine-targeted therapies with traditional surgical approaches, explore additional cytokines, such as IL-1β, IL-10, and NGF, to better understand the inflammatory pathways involved in meniscus-related pain and degeneration, and assess the potential of cytokine levels as predictive biomarkers for pain severity and therapeutic outcomes.

Limitations

The present study had several limitations. First, the study has a very small sample size, which may limit the generalizability of the results. The study design is observational, limiting the ability to establish causality. Second, only three proinflammatory cytokines were quantitatively analyzed in this study. Other inflammatory mediators, such as IL-1, IL-8, IL-10, MMP-1, MMP-3, and MMP-13 [22], should be evaluated in future studies. Third, proinflammatory cytokines in the synovial fluids and membranes were not evaluated. Since the increase in inflammatory cytokines in synovial fluid and synovial tissue can also contribute to knee pain, it was necessary to collect samples and measure these data during surgery to evaluate their potential as confounding factors in the results. Future studies should evaluate the correlation between the results of the present study and the results obtained from synovial fluids and synovial membranes. Finally, an HLM in the ipsilateral knee was used as the control in this study. However, no published data were identified that used healthy medial menisci on the contralateral side for comparison. While the types of cytokines examined have been covered in other studies**, **research specifically investigating inflammatory cytokines within injured meniscal tissue remains extremely limited and we believe that the findings of this study will provide valuable insights and serve as a significant reference for future research exploring the relationship between the meniscus and pain.

## Conclusions

The results of the study confirmed a local increase in inflammatory mediator levels in the injured medial meniscal tissue in patients with MMPRT; specifically, the levels of TNF-α and IL-6 increased significantly at the site of the meniscal tissue injury. It was suggested that these inflammatory mediators (TNF-α and IL-6) within the IMM may be associated with preoperative pain in patients with SIF in the MFC caused by MMPRT. NGF was undetectable in the meniscal tissue, suggesting that further studies should explore other potential inflammatory mediators or experimental designs to assess NGF's role in meniscal injury. This study provides new insights into the role of TNF-α and IL-6 in the pathophysiology of medial meniscal injury and subchondral insufficiency fractures, which may have important implications for pain management and therapeutic targeting. Further evaluation of the biological effects of meniscal injury is warranted to establish a treatment protocol for pain relief.
